# Individual variation in fresh and frozen semen of Bali bulls (*Bos sondaicus*)

**DOI:** 10.14202/vetworld.2020.840-846

**Published:** 2020-05-05

**Authors:** R. Indriastuti, M. F. Ulum, R. I. Arifiantini, B. Purwantara

**Affiliations:** 1 Study Program of Reproductive Biology, Graduate School, IPB University, Bogor, Indonesia; 2 Department of Veterinary Clinic, Reproduction and Pathology, Faculty of Veterinary Medicine, IPB University, Bogor, Indonesia

**Keywords:** Bali bull, individual factors, sperm quality

## Abstract

**Aim:**

This study aimed to analyze the individual factors influencing the sperm quality of Bali bulls at Baturiti Artificial Insemination (AI) center.

**Materials and Methods:**

Semen that was ejaculated from nine Bali bulls was collected using artificial vaginas (n=5/bull). Semen ejaculates were evaluated immediately after collection to measure the quality of the fresh semen, including semen volume, sperm concentration, progressive motility, membrane integrity (MI), and abnormal morphology. Frozen semen was evaluated for progressive sperm motility, concentration, viability, MI, abnormal morphology, and deoxyribonucleic acid (DNA) fragmentation. Other secondary data, focusing on semen quantity (semen volume and sperm concentration), were also collected from frozen the semen production data of the Baturiti AI center from 2017 to 2019. Data were analyzed statistically using a completely randomized design, and one-way analysis of variance was applied to find differences among individual bulls.

**Results:**

Significant differences (p<0.05) were found among the bulls in semen volume, sperm motility, concentration, and MI of the fresh semen. Significant differences (p<0.05) were also found among the bulls in sperm motility, viability, MI, abnormal morphology, and DNA fragmentation of the frozen semen.

**Conclusion:**

Individual variation in all the tested sperm parameters of the fresh semen of Bali bulls, except sperm viability and abnormalities, was noted. Similarly, individual variation in all the tested sperm parameters in frozen semen, except sperm concentration, was noted. Therefore, individual factors can be used for selecting a superior bull in Bali cattle.

## Introduction

Bali cattle are a native Indonesian breed that has many advantages over other cattle. They easily to adapt to various types of feed, environmental conditions, and extreme climate changes, and they have good reproductive efficiency [1]. For beef production, Bali cattle have a fairly high percentage yield of carcass at 51% [2] to 55.61% [3]. The value of service per conception (S/C) is 1.76-1.84 [4,5], with the value of conception rate (CR) of 80.00-86.67% [6]. The reproductive performance of bulls can be assessed from their semen quality. The quality of semen is influenced by various factors, including environmental factors such as the season [7-9], relative humidity [10], temperature [7], feed [11], and genetic factors [12]. The variation of individual bulls is one aspect of genetic factors. Research regarding the influence of individual factors on the quality of semen has been widely reported for bulls [13], stallions [14], bucks [15], rams [16], and elephants [17].

Good semen quality is necessary for increasing livestock populations, including Bali cattle. The breeding of Bali cattle in Bali Province is conducted through artificial insemination (AI) using frozen semen. The frozen semen that is used is only derived from bulls in the Baturiti AI center. It is produced according to the standards set by the Indonesian National Standard (SNI) of bull frozen semen number 4869.1:2017, which specifies a value of post-thawing motility of >40% with individual scores >2 and the concentration of sperm >25 × 10^6^ AI/dose [18]. A potentially fertile sperm is considered to be a living and motile sperm that has a normal morphology and intact chromatin [19]. The parameters of sperm viability, motility, and abnormalities are not sensitive enough to predict bull fertility. Rather the main factor influencing successful fertilization is the ability of chromatin to form the pro-nucleus after fertilization. According to Tsakmakidis *et al*. [20], only sperm with normal chromatin structure is able to fertilize the oocyte the sperm – zona pellucida binding process. Chromatin serves to package deoxyribonucleic acid (DNA) in the sperm nuclei into more dense and compact shape [21]. The packaging of sperm DNA protects it and prevents DNA fragmentation during the transportation process from the cauda epididymis to the fertilization site in the female reproductive tract. A specific nucleic protein called protamine (PRM) is needed to build a chromatin structure, and this protein replaces histone during the process of spermiogenesis. The presence of protamine is used as an indicator of chromatin maturity. This is useful because sperm with unripe chromatin conditions will cause fragmented DNA, and the occurrence of DNA fragmentation causes low fertilization rates [22].

Considering the Baturiti AI center is the only producer of frozen semen from Bali bulls for the AI program in the Bali Province, it is important to evaluate the productivity of Bali bulls regularly through the evaluation of the fresh and frozen semen quality of each bull. This research aims to analyze the influence of individual factors on the semen quality of Bali bulls in the Baturiti AI center.

## Materials and Methods

### Ethical approval

This research was carried out following standard operational procedure SNI ISO 9001:2015 No. 824 100 15084 at the Baturiti AI Center and supervised by a Veterinarian from this institution. Baturiti AI center Ethical Committee provided ethical guidance and approval on the responsible conduct of the use of bull for semen collection.

### Experimental animals

This research was conducted in the Laboratory of Semen Processing at the Baturiti AI center, Tabanan, Bali, and the Laboratory of Reproductive Rehabilitation Unit, Faculty of Veterinary Medicine, IPB University, Bogor, West Java, Indonesia. Nine Bali bulls of productive age (3-11 years old), namely, Mertasari, Blandar, Bugamanta, Bangkardi, Buwana Merta, Bangtidar, Bulbakanta, Busada Merta, and Budaparta, were used in this research at the Baturiti AI center. All the experimental animals were fed 50 kg of forages, 5 kg of concentrate feed, and water *ad libitum*. Semen ejaculates were collected twice a week using artificial vaginas. A day before semen collection, all the experimental animals were exercised. Five ejaculates were collected from each bull. Only semen ejaculates with more than 70% sperm motility, more than 800 × 10^6^/mL sperm concentration, and <20% sperm abnormalities were used. Semen collection was carried out from March to April 2019.

### Evaluation of fresh semen quality

Semen ejaculates were evaluated for semen volume, progressive sperm motility, viability, membrane integrity (MI), and abnormal morphology immediately after collection. Other secondary quantitative data of semen volume and sperm concentration, from the 2017 to 2019 frozen semen production data of the Baturiti AI center were also used.

Progressive sperm motility was evaluated using the Computer-Assisted Semen Analysis (CASA) software (Sperm Vision Minitube version 3.5.6.2, Germany) [23]. For each semen sample, 10 μL of semen was taken using a micropipette and mixed with 40 μL of saline solution over a glass slide, which was closed with a glass cover. Observations of sperm motility were conducted under the microscope with 200×, and as many as seven fields were used with a value ranging from 0% to 100%.

The concentration of sperm was measured using an SDM 6 photometer (Minitube, Germany) [24]. A total of 35 µL of fresh semen were inserted into a special cuvette containing 3.5 mL of saline solution (1:100). Then, the cuvette was closed with parafilm plastic and homogenized. After this, the cuvette was placed on the photometer, and then, the bull’s identity and the percentage sperm motility were inputted into the photometer before running the program according to the manual.

Sperm viability was analyzed using the eosin-nigrosin staining method [25]. A total of 5 μL of semen were ripped over a glass slide, 20 μL of eosin-nigrosin was added, and the mixture was then homogenized. The mixture was smeared on an object slide, dried above a heating table, and observed under a microscope with 400× in 10 fields. The living sperm showed a clear-colored head, whereas the dead sperm showed a purplish-red head.

The integrity of the sperm plasma membrane was observed using a hypo-osmotic swelling (HOS) solution test [25]. A total of 20 μL of semen were mixed into a microtube containing 1000 μL of HOS solution, and then, this was homogenized. The microtube was incubated at a temperature of 37°C for 30 min. A total of 10 μL of suspension were ripped over a glass slide and covered with a glass cover, and then, it was observed under the microscope with 400× at 10 points of view. Sperm with an intact plasma membrane showed the reaction of a coiled tail.

The evaluation of sperm morphology was conducted using the Williams staining following the method of Kavak *et al*. [26] with modification. For each sample, 10 µL of fresh semen diluted with saline solution (1:4) was dropped on the surface of a glass slide, and then, a reviewing slide was prepared. The slide was later fixed over the flame of a Bunsen burner, washed using an absolute alcohol solution for 4 min and air-dried. It was repeatedly dipped into 0.5% chloramine solution for 2 min to remove the mucus in the review, then washed with distilled water, followed by 95% alcohol and stained with a William’s solution for 6 min. The slide was washed with flowing water, dried, and then observed under a microscope with 400×. Observations of sperm abnormalities were performed on as many as 500 sperms.

### Evaluation of frozen semen quality

Frozen semen from four batches of ejaculate production with five straws/bull/ejaculate was used. The frozen semen was thawed for 30 s at a temperature of 37°C, and it was transferred into microtubes. The microtubes were incubated in a water bath at 37°C during evaluation. The evaluation parameters of sperm motility, viability, plasma MI, and sperm abnormalities were measured using the same approach as for the fresh semen, with a few modification: The progressive sperm motility assessment was conducted without saline solution, sperm viability was measured using only 10 µL eosin-nigrosin solution, sperm concentration was measured using a Neubauer hemocytometer [27], sperm morphology was evaluated using 2% chloramine solution, and sperm DNA fragmentation was assessed using a Halomax Kit^®^ following García-Macías [28] with modifications in the stage of dehydration and the staining of samples. A total of 15 µL of frozen semen was diluted in the 85 µL of phosphate buffer saline solution until the sperm concentration was 15 million sperm/mL, and then, 25 µL of the semen solution was added to 50 µL of liquid agarose at a temperature of 37°C. Next, 25 µL of the suspension was dropped on the slides, which were refrigerated at 4C, then covered with a cover glass and allowed to stand for 5 min. The cover glass was then opened carefully, and the slides were trimmed with 10 mL lysis solution and incubated at room temperature for 4 min. The slides were then washed with distilled water for 5 min and then dehydrated in 70% and 100% ethanol solution, respectively, for 2 min. The slides were colored using Wright’s eosin methylene blue solution and air-dried. Samples were observed under a light microscope with 400×. The integrity of the sperm chromatin was used as an indicator of DNA fragmentation. The sperm nuclei with intact DNA showed a small and compact halo, whereas a large and diffuse halo was signs of fragmented sperm DNA.

### Statistical analysis

Means and standard errors of means were calculated for all parameters. Statistical differences among bulls were obtained using one-way analysis of variance with Duncan’s multiple range test (SPSS 21.0) and p<0.05 was considered statistically significant [29].

## Results

### Individual variation in the quality of fresh semen

Individual variation was found in semen volume (6.32±0.07 mL), sperm motility (89.62%±0.51%), sperm concentration (1164.81±9.10×10^6^/mL), and plasma MI (81.54%±0.54%) of the fresh semen of the nine Bali bulls (Table-1). However, no significant variation was found in sperm viability (82.53%±0.44%) or sperm abnormalities (4.71%±0.19%). The highest semen volume was found for Bulbakanta (8.50 mL) and Busada Merta (8.12 mL), moderate semen volume was found for Buwana Merta, Mertasari, Bangkardi, and Bugamanta (5.30-7.40 mL), and Blandar and Budaparta showed the lowest semen volume (4.56 and 4.63 mL). However, the individuals with the highest sperm volume did not necessarily have the highest sperm motility. The highest percentage of sperm motility was found for Bugamanta and Bangkardi (91.81%-92.53%), and the lowest percentage sperm motility was found for Mertasari dan Bulbakanta (86.79-86.92%). Buwana Merta, Blandar, Budaparta, Bangtidar, and Busada Merta demonstrated moderate sperm motility (89.19-91.57%). The individuals with moderate sperm motility showed the highest sperm concentrations. Buwana Merta had the highest sperm concentration (1492.67×10^6^/mL), followed by moderate sperm concentration found for Busada Merta (1312.27×10^6^/mL), Bangtidar (1277.32×10^6^/mL), Mertasari (1233.75×10^6^/mL), Budaparta (1204.01×10^6^/mL), and Blandar (1114.05×10^6^/mL). The lowest sperm concentration was found for Bugamanta, Bangkardi, and Bulbakanta (942.42-964.12×10^6^/mL). The highest value of sperm MI was shown by Blandar, followed by Bangtidar, Budaparta, Bangkardi, Mertasari, Bulbakanta, Busada Merta, and Bugamanta. Buwana Merta showed the lowest percentage of sperm MI.

**Table-1:** Quality of fresh semen in various individuals of Bali bull.

Bulls	Volume (mL)	Sperm motility (%)	Sperm concentration (10^6^/mL)	Sperm viability (%)	Sperm membrane integrity (%)	Sperm abnormalities (%)
Mertasari	6.57±0.11^c^	86.79±1.56^c^	1233.75±18.18^c,d^	81.74±1.75	81.88±1.58^b,c,d,e^	5.00±0.37
Blandar	4.56±0.12^e^	90.53±0.68^a,b,c^	1114.05±17.21^e^	83.17±1.60	86.22±0.82^a^	4.80±0.80
Bugamanta	5.30±0.15^d^	91.81±1.46^a^	941.42±20.01^f^	82.40±0.90	78.40±1.70^e^,^f^	4.80±0.56
Bangkardi	6.17± 0.13^c^	92.53±0.64^a^	949.60±15.60^f^	82.53±1.95	82.13±0.55^b,c,d^	5.20±0.67
BuwanaMerta	7.40±0.21^b^	91.57±0.95^a,b^	1492.67±20.64^a^	80.06±2.99	77.88±1.64^f^	4.90±0.64
Bangtidar	5.65±0.12^d^	89.19±0.78^a,b,c^	1277.32±18.66^b,c^	80.92±1.59	84.41±0.54^a,b^	4.35±0.28
Bulbakanta	8.50±0.23^a^	86.92±1.47^c^	964.12±24.19^f^	81.95±1.91	80.28±1.54^c,d,e,f^	4.95±0.54
BusadaMerta	8.12±0.21^a^	89.43±0.96^b,c^	1312.27±18.40^b^	79.62±2.50	78.72±0.98^d,e,f^	4.95±0.38
Budaparta	4.63±0.09^e^	89.62±0.51^a,b,c^	1204.01±22.62^d^	82.09±1.34	82.73±0.32^a,b,c^	3.45±0.79
Mean	6.32±0.07	89.62±0.51	1164.81±9.103	82.53±0.44	81.54±0.54	4.71±0.19

Data show all mean±SEM (n=5). Means in a column with different superscripts a, b, c, d, e, and f differ significantly at p<0.05. SEM=Standard error of means

### Individual variation in the quality of frozen semen

Individual variation was found in sperm motility (69.37%±0.41%), viability (77.57%±0.25%), MI (72.83%±0.33%), abnormalities (6.50%±0.23%), and DNA fragmentation (3.00%±0.29%) of frozen semen (Table-2). However, no significant variation was found in sperm concentration per straw (27.02%±0.21×10^6^/dose).

**Table-2:** Quality of frozen semen in various individuals of Bali bull.

Bulls	Sperm motility (%)	Sperm concentration (10^6^/dose)	Sperm viability (%)	Sperm membrane integrity (%)	Sperm abnormalities (%)	Sperm DNA fragmentation (%)
Mertasari	67.59±1.01^ab^	27.28±0.92	76.30±0.28^c^	69.14±1.15^d^	7.80±1.29^a^	2.64±0.15^b,c^
Blandar	70.66±1.99^a^	26.76±0.45	79.92±0.84^a^	77.09±0.58^a^	7.15±0.81^a^	2.50±0.00^b,c^
Bugamanta	71.23±1.10^a^	26.03±0.69	77.60±1.06^a,b,c^	74.42±0.40^a,b^	6.60±1.31^a,b^	2.89±0.68^b,c^
Bangkardi	68.21±1.61^a,b^	26.76±0.73	75.79±0.84^c^	73.30±0.91^b,c^	7.80±0.59^a^	4.79±0.87^a^
BuwanaMerta	69.57±1.07^a^	27.66±0.59	78.98±0.64^a,b^	72.83±0.79^b,c^	5.50±0.76^b,c^	1.64±0.15^b,c^
Bangtidar	64.69±1.82^b^	26.41±0.60	76.92±0.53^b,c^	72.51±1.37^b,c^	5.55±0.84^b,c^	1.54±0.24^c^
Bulbakanta	70.84±0.61^a^	27.92±0.42	76.88±0.62^b,c^	71.12±0.82^c,d^	7.50±0.62^a^	3.58±0.96^a,b^
BusadaMerta	70.37±0.97^a^	27.33±0.54	78.97±1.11^a,b^	74.44±0.98^a,b^	6.45±0.91^a,b^	2.64±0.18^b,c^
Budaparta	68.31±1.02^a,b^	27.11±0.59	77.84±0.77^a,b,c^	68.58±0.86^d^	4.15±0.93^c^	1.24±0.24^c^
Mean	69.37±0.41	27.02±0.21	77.57±0.25	72.83±0.33	6.50±0.23	3.00±0.29

Data show all mean±SEM (n=5). Means in a column with different superscripts a, b, c, and d differ significantly at p<0.05. SEM=Standard error of means

The highest percentage of sperm motility (69.57%-71.23%) was found for Buwana Merta, Busada Merta, Blandar, Bulbakanta, and Bugamanta. This was followed by moderate sperm motility shown by Mertasari (67.59%), Bangkardi (68.12%), and Budaparta (68.13%), and the lowest sperm motility was found for Bangtidar (64.69%). The bull with the highest percentage of sperm viability was Blandar (79.92%), whereas the lowest percentage was found for Bangkardi (75.79%) and Mertasari (76.30%). A total of six bulls showed moderate sperm viability (76.88%-78.98%). The highest percentage MI was found for Blandar (77.09%). Busada Merta (74.44%), Bugamanta (74.42%), Bangkardi (73.30%), Buwana Merta (72.83%), Bangtidar (72.51%), and Bulbakanta (71.12%) showed moderate values for sperm MI, and the lowest percentage was found for Mertasari and Budaparta (69.14% and 68.58%). The highest percentage of sperm abnormalities was shown by Mertasari and Bangkardi (7.80%) followed by Bulbakanta (7.50%) and Blandar (7.15%). Buwana Merta, Bangtidar, Busada Merta, and Bugamanta represented moderate sperm abnormalities (5.50%-6.60%) and the lowest percentage was found for Budaparta at only 4.15%. The bull with the highest percentage of sperm DNA fragmentation was Bangkardi (4.79%), whereas the lowest values were in Budaparta (1.24%) and Bangtidar (1.54%).

## Discussion

### Individual variation in the quality of fresh semen

The finding that there is individual variation in semen volume is similar to the findings of research by Marques *et al*. [30] on ram semen from numerous individuals. The variation in semen volume among bulls can be influenced by the condition of the male reproductive organs and accessory sex gland secretions. Semen is composed of sperm and plasma seminal fluid. Setchell [7] reported that sperm makes up only 10% of the semen volume, whereas the rest (90%) is the seminal plasma. Ndovi [31] stated that accessory gland secretions play an important role in influencing ejaculate volume. Seminal plasma consists of secretions from the urethral and bulbourethral gland (<10%), the prostate gland (20-40%), the testicular-epididymis (<10%), and the seminal vesicle (50-80%).

The values found for the sperm motility of all the bulls (Table-1) were higher than those found by Haryani *et al*. [32] and Nabilla *et al*. [23], and individual variation in sperm motility has also been reported by Marques *et al*. [30] for ram fresh semen. Such variation in sperm motility among individuals can be caused by variations in mitochondrial function in generating an energy substrate in the form of adenosine triphosphate (ATP). Amaral *et al*. [33] reported that the mitochondrial activity of sperm is influenced by the membrane mitochondria potential, mitochondrial enzyme activity, mitochondrial volume, oxygen consumption, and mitochondrial respiration.

Individual variation in sperm concentration has also been reported by Narwade *et al*. [9] for buck semen. The range in sperm concentration found for Bali bulls in this study is comparable to that found by Nabilla *et al*. [23]. Variation in sperm concentration can be caused by the number of spermatogenic cells and their activity in the testes to develop sperm through the process of spermatogenesis. Setchell [7] stated that the production of sperm depends on the size of the testes hormones and environmental temperature.

The lack of individual variation in sperm abnormalities in fresh semen is in accordance with similar research on the Egyptian bull [13] and Saloia ram [30]. Sperm abnormalities might occur as the result of disturbances in the stage of spermatogenesis or the maturation process in the epididymis. Al-Makhzoomi [34] states that abnormal spermiogenesis causes abnormalities in the sperm head resulting from a defect in chromatin condensation. Disturbances in the maturation of sperm in the epididymis may result in immature sperm that is identified by the presence of a proximal cytoplasmic droplet. The range of sperm abnormalities found in this research is similar to the percentage of total sperm abnormalities in Friesian Holstein bulls reported by Purwantara [35]. Our finding of a lack of individual variation in the sperm viability of Bali bulls is in contrast to findings reported by Narwade *et al*. [9] that there is individual variation in the sperm viability of buck semen. In addition, the value of sperm viability obtained in this study was higher than that reported by Lukman *et al*. [36].

Results obtained for sperm MI were higher than those reported by Hapsari *et al*. [37]. Differences in the proportion of cholesterol in the sperm membrane might cause variation in sperm MI. According to Sheriff and Ali [38], the presence of cholesterol, which is an amphipathic molecule, affects MI. This result is also in accordance with the study reported by Narwade *et al*. [9].

Similar to our findings of individual variation in all parameters except for sperm viability and abnormalities, individual variation in semen volume, sperm motility and viability, mass movements, sperm concentration, membranes integrity, acrosome integrity [9], and sperm abnormalities [39] has been found in goats as well as in boar sperm motility, MI, and mitochondrial activity [40].

### Individual variation in the quality of frozen semen

Individual variation in sperm motility in frozen semen has also been found for Egyptian bulls [13] and in Saloia rams [30]. The range of sperm motility that was obtained in this study indicates that all bulls had sperm with good freezability. This might be influenced by the ability of sperm membranes to protect components of the entire cell and respond to extracellular osmotic pressure during cryopreservation. According to Khalil *et al*. [41], sperm plasma membranes are responsible for the regulation of the calcium, potassium, and sodium ions that are needed for mitochondrial activity and sperm motility. Differences in the morphology of sperm mitochondria also affect the alteration of mitochondrial structure during cryopreservation, which might lead to decreased mitochondrial function in producing ATP for sperm motility. Khalil *et al*. [39] also stated that after freezing, the mitochondrial morphology undergoes structural changes and decreased function by 15%.

Although no variation was found in the sperm concentration of frozen semen, the concentrations met the standards of the SNI 4869.1:2017, which is a sperm concentration of > 25 million sperm/dose (BSN, 2017). The findings also indicate that the production of frozen semen at the Baturiti AI center has been conducted precisely, and therefore, consistent sperm concentrations were obtained at each dose.

The sperm viability findings for frozen sperm are similar to those of Mohammed and Ahmed [13] for pure Egyptian bulls, Marques *et al*. [30] for Saloia rams, and Lukman *et al*. [36] for Bali bulls. The individual variation in sperm viability found in this study might be caused by the proportion of lipid and cholesterol in the sperm membrane. The proportion of lipid and cholesterol influences the alteration of membrane structure, including sperm heads after freezing. In a previous study, about 18% of the sperm population showed the alteration of sperm head membranes during cryopreservation [41].

Individual variation in sperm MI has also been found in pure Egyptian bulls [39]. Variations in sperm with MI are caused by sperm membrane damage during cryopreservation process. Cryopreservation process affects the capability of membranes to respond to osmotic pressure. Sperm membrane damage decreases the motility and viability of sperm and even causes cell death. According to Khalil *et al*. [41], about 15-20% of post-thawing sperm damage to plasma membrane leads to the alteration of potassium, calcium, and sodium ions that are responsible for sperm motility and mitochondrial activity.

The finding of individual variation in sperm abnormalities in frozen semen is in disagreement with Mohammed and Ahmed [13] and Marques *et al*. [30] who found no individual variation in sperm abnormalities in pure Egyptian bulls and Black Bengal bucks, respectively. The rate of sperm abnormalities obtained in this study was lower than that found by Carreira *et al*. [42]. Such differences in sperm abnormalities might be caused by different survival rates of sperm during the cryopreservation process. According to Loomis and Graham [14], the ability of sperm to survive during the cryopreservation process differs between individuals in species. Differences might influence the ability of sperm to survive during the cryopreservation process in the biochemical and metabolic properties of sperm cells. Indeed, in a previous study, the cryopreservation process caused an increase in the defect of the sperm head, midpiece, and tail [43].

The levels of sperm DNA fragmentation found were lower than that found by Dogan *et al*. [21]. This might be caused by internal factors such as spermatogenesis impairment or external factors such as semen handling and processing. Spermatogenesis impairment might lead to imperfections in chromatin maturation. The process of chromatin maturation involves the replacement of specific proteins from histone to protamine. Protamine plays an important role in forming a condensed and compact chromatin structure to maintain the stability of DNA. Fortes *et al*. [44] have defined that sperm protamine deficiency causes DNA damage.

**Figure-1:**
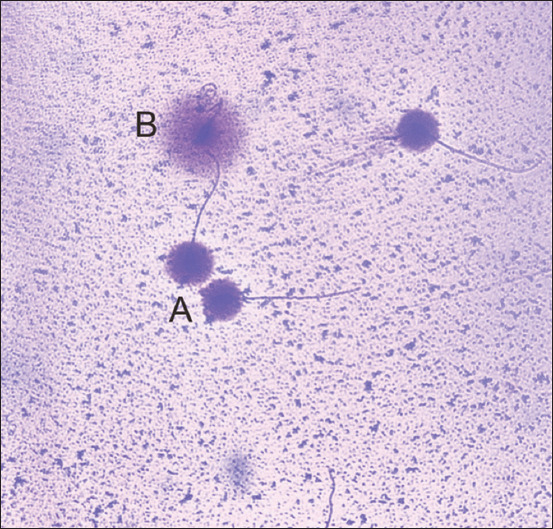
Halomax^®^ sperm DNA fragmentation test results. Sperm with (A) no fragmentation DNA (intact and small halo) and (B) fragmented DNA (large and dispersed halo).

Sperm with fragmented DNA is characterized by the formation of large and dispersed halo formed due to the nucleic proteins releasing the DNA fragments of two broken strands around the chromatin and forming a peripheral halo (Figure-1). This finding was similar to García-Macías *et al*. [28] and Dogan *et al*. [21]. Sperm with fragmented DNA will interfere with the sperm function during fertilization and development, and therefore, they can be used as a parameter to predict bull fertility [22].

Individual variation in all parameters except for sperm concentration is similar to findings for individual variation in bull sperm motility, viability, abnormalities, MI, and acrosome integrity of frozen semen [13]. In goats, individual variation was found in sperm motility and viability in buck semen [9].

Therefore, based on our findings, individual variation in Bali bulls in fresh and frozen semen can be used for selecting and determining of bull rankings in the Baturiti AI center. Bull rankings can be determined according to semen quality. There are at least three parameters of semen quality needed to determine the level of productivity of bull frozen semen, namely, semen volume, sperm concentration, and motility. These parameters are used to predict the productivity of frozen semen of each bull per year. The superior Indonesian local breed bull should have > 7500 straws/bull/year frozen semen production [45].

## Conclusion

This study found that individual variation in Bali bull fresh semen can affect all sperm parameters, except sperm viability and abnormalities, and individual variation in frozen semen can affect all sperm parameters except for sperm concentration. Thus, it can be concluded that individual factors can be used to select a superior bull in Bali cattle.

## Authors’ Contributions

BP designed the general concept and supervised the management of the experiment. RI and RIA designed the questionnaire and carried out the data collection. RI, RIA, and MFU analyzed the data. RI and RIA drafted the manuscript. BP revised and finalized the manuscript. All authors read and approved the final manuscript.
